# Nutrition literacy is associated with general obesity, abdominal obesity, and body fat percentage obesity

**DOI:** 10.3389/fnut.2025.1555725

**Published:** 2025-03-12

**Authors:** Yan Cui, Qi Qi, Yuhui Sun, Rumeng Liu, Wending Yin, Huaqing Liu

**Affiliations:** ^1^School of Public Health, Bengbu Medical University, Bengbu, Anhui, China; ^2^Huainan Maternal and Child Health and Family Planning Service Centre, Huainan, Anhui, China

**Keywords:** nutrition literacy, general obesity, abdominal obesity, body fat percentage, adults

## Abstract

**Background:**

Obesity is associated with unhealthy eating behavior among adults. Nutrition literacy (NL) is an important determinant of eating behavior. This study investigated the association of NL with general obesity, abdominal obesity, and body fat percentage (BFP) obesity among adults.

**Methods:**

This study was a cross-sectional survey on the Nutrition Literacy and Obesity, conducted in Bengbu City, China (May to July 2023). The Short-Form Nutrition Literacy scale was used to evaluate the NL of adults. General obesity was defined as a body mass index of ≥28 kg/m^2^. Abdominal obesity was defined as a waist circumference of ≥90 cm in men and ≥85 cm in women. BFP obesity was defined as a BFP of ≥30% in men and ≥42% in women. Binary logistic regression analysis was performed to identify the correlations of NL with general obesity, abdominal obesity, and BFP obesity in adults. Subgroup analyses and interaction tests were also performed.

**Results:**

Participants with a high level of NL had low odds of general obesity (odds ratio [OR]: 0.66; 95% confidence interval [CI]: 0.43–0.99), abdominal obesity (OR: 0.63; 95% CI: 0.46–0.87), and BFP obesity (OR: 0.55; 95% CI: 0.35–0.87). In subgroup analyses and interaction tests, age significantly influenced the negative correlations of NL with abdominal obesity and BFP obesity (*p* for interaction <0.05), but not general obesity (*p* for interaction >0.05). Moreover, sex and smoking status significantly influenced the negative correlations of NL with general obesity and abdominal obesity (*p* for interaction <0.05), but not BFP obesity (*p* for interaction >0.05), and drinking status significantly influenced the negative correlations of NL with abdominal obesity (*p* for interaction <0.05), but not general obesity and BFP obesity (*p* for interaction >0.05). However, marital status did not significantly influence the correlation of NL with obesity (*p* for interaction >0.05).

**Conclusion:**

Our findings highlight that adults with high levels of NL have low odds of general obesity, abdominal obesity, and BFP obesity. Age, sex, smoking status, and drinking status influence the correlation between NL and obesity. The results indicate the importance of NL in the prevention and management of obesity in adults. To effectively address the challenges of adult obesity management, public health practitioners should tailor nutrition education and skill training programs to specific demographic profiles.

## 1 Introduction

Obesity is a major cause of chronic diseases and mortality in adults (1). The global prevalence of obesity has increased in recent decades, with the number of people with obesity almost tripling since 1975 (2). In 2014, China reported the highest number of adults with obesity (3). National estimates indicate that 34.3% of all Chinese adults are overweight and that 16.4% are obese (4). These estimates indicate that more than half of all adults in China are either overweight or obese (5). The prevalence of obesity is increasing. By 2030, the prevalence of overweight and obesity among Chinese adults will reach 65.3% (6). The increasing prevalence of obesity is imposing high economic and health-care burdens, with clinical data indicating a strong positive correlation between obesity and annual personal health-care expenditure (7). The high prevalence of obesity poses major challenges to the public health and economic stability in China.

Diet and nutrition are key modifiable factors in the management of obesity (8). The aetiology of obesity is multifaceted and involves a confluence of genetic, behavioral, dietary, psychological, economic, social, biomedical, and environmental determinants (9). Dietary control is the most rapid and convenient approach for mitigating and managing obesity (10). Empirical evidence suggests that restricting the consumption of high-calorie foods, such as foods rich in sugars and fats, is vital for the prevention and treatment of obesity (11). However, the complex, ever-evolving nature of the current food environment makes it difficult for individuals to obtain scientifically accurate health information (12), reducing their ability to select healthier dietary options. Consequently, fostering competence and literacy in this domain is paramount. Nutrition literacy (NL) as an emerging field of study, where an individual is capable of acquiring, understanding, and applying a wide range of complex nutrition-related information (13). It indicates not only individuals’ foundational knowledge of nutrition but also their adeptness at the practical application of this knowledge. Demographic studies have consistently revealed that individuals with overweight or obesity typically have limited knowledge of nutrition, which reduces their ability to make appropriate dietary choices; this, in turn, perpetuates problems related to overweight and obesity (14). NL plays a pivotal role in shaping healthy dietary habits, which could influence weight and fat distribution.

Most studies on the nutritional aspect of obesity have focused on the correlations between individual nutrients and biochemical indicators (15). However, very few studies have investigated the role of NL in obesity. Rosenbaum et al. (16) reported that NL influences weight loss behaviors and skills, such as monitoring calorie intake. A cross-sectional study conducted in Chongqing, China, revealed a negative association between NL and overweight/obesity in individuals aged 10–18 years (17). The concepts of NL and health literacy overlap (18, 19). Thus, studies on the correlation between health literacy and obesity may provide insights into the correlation between NL and obesity. Zare-Zardiny et al. (20) found no statistically significant correlation between body mass index (BMI) and health literacy in 15–19-year-old high-school students. However, a population-based study involving 11–12-year-old school children in Taiwan reported a strong correlation between health literacy and obesity; the results revealed that children with a higher level of health literacy had a lower risk of obesity (21). In an exploratory study, Sharif and Blank (22) demonstrated that health literacy was negatively associated with BMI in individuals aged 6–19 years. NL can be developed, internalized, and applied to daily life for long-term benefits (23). Therefore, the correlation between NL and obesity in adults should be explored to provide a theoretical basis and inform practical strategies for obesity prevention and management.

Limited attention has been paid to the correlation between NL and obesity, particularly in adults. Obesity is characterized by excessive accumulation of body fat, with multiple organ-specific consequences (24). Although BMI is the most common measure of obesity in epidemiological studies (25), it only reflects overall obesity and not body fat distribution (26). Emerging evidence suggests that body fat distribution is associated with the risks of obesity and mortality (27). Fat distribution is typically determined by measuring waist circumference (WC) (28), and body fat percentage (BFP) is used to diagnose obesity, which reflects the amount of body fat (29). High NL fosters healthy eating behavior (30), thereby reducing calorie intake, body weight, and obesity risk and optimizing diet (31). Adults with higher NL levels tend to make healthier dietary choices; thus, children under the care of adults with high NL levels have a high likelihood of maintaining a healthy body weight (32). By investigating the correlation between NL and obesity and by improving NL in adults, population-wide obesity can be reduced and thus public health can be enhanced. The present study explored the correlations between NL and general obesity, abdominal obesity, and BFP obesity in adults. This study can serve as a theoretical basis and inform practical strategies for preventing and managing obesity in adults.

## 2 Methods

### 2.1 Data source and study population

This study was a cross-sectional survey on the Nutrition Literacy and Obesity, which was conducted between May and July 2023 in Bengbu City, China. The stratified multistage random sampling method was used to select participants for the survey. In the first stage, two urban areas and two rural counties were randomly selected from Bengbu City as the primary sampling sites. In the second stage, two streets and two villages were randomly selected from each of the two urban areas and two rural counties to serve as specific sampling points. In the third stage, 110 households were randomly selected from the designated streets and villages. Within these households, all individuals who met the survey criteria were considered to be respondents. The inclusion criteria for participants were (1) they must be permanent adult residents, and (2) they should have no language barriers that would impede communication, and (3) they did not receive nutrition and/or food literacy training previously. The exclusion criteria included: (1) individuals suffering from a severe physical illness that would prevent them from participating in the survey and (2) those who decline to participate in the survey. After obtaining informed consent from the participants, a trained investigator conducted face-to-face interviews with them, following a standardized protocol. Of the 2,279 questionnaires collected, 123 participants were excluded from the study because of incomplete data on NL, height, weight, waist circumference, and age. Thus, the final sample comprised 2,156 valid responses (validity rate: 94.60%). This study was approved by the Ethics Committee of Bengbu Medical University.

### 2.2 Sample size calculation

The following formula was adopted to estimate the sample size:


$$N=\frac{Z_{\alpha }^{2}P\left(1-P\right)}{\delta^{2}}$$

*Z*_0.05_ = 1.96, predicted by an adult obesity rate (*P*) of 14%, *δ* = 0.15 × *P*. The research was divided into two strata according to urban and rural areas, with a calculated sample size of 2098. A total of 2,156 valid responses were obtained in this study, thereby fulfilling the analytical requirements with an adequate margin.

### 2.3 Assessment of NL

Nutrition literacy was assessed using the 12-item Short-Form Nutrition Literacy scale (NL-SF12), which was developed in another study (33). The NL-SF12 scale was derived from the original 43-item scale used for assessing NL in adults (34). The NL-SF12 scale, which exhibits a Cronbach’s *α* value of 0.881, exhibited high internal consistency in the present study, indicating that its items effectively covered various aspects of NL. This scale assesses NL in two primary domains: cognition and skills. The NL-SF12 scale comprises two subscales: nutrition cognition, which includes four items, and nutrition skills, which includes eight items. In total, it consists of 12 items. Responses are rated on a 5-point Likert scale, with end points ranging from 1 (*strongly disagree*) to 5 (*strongly agree*). The total score is calculated by summing the scores for the two domains and the overall scores. Higher scores indicate higher NL levels. For this study, NL levels were categorized into the following quartiles: Q1, low NL levels; Q2, low–moderate levels; Q3, high–moderate NL levels; and Q4, high NL levels.

### 2.4 Assessment of obesity

Weight, height, and waist circumference (WC) were measured according to anthropometric measurements method in health surveillance (35) by trained investigators. A Meilen height and weight scale MSG008 was used to record height (cm) and weight (kg), with an accuracy of 0.1 kg and 0.1 cm, respectively. A soft tape measure was used to record WC (cm) with an accuracy of 0.1 cm. BMI, WC, and BFP were used to diagnose general obesity, abdominal obesity, and BFP obesity, respectively. BMI was calculated using the following formula: height (cm)/weight^2^ (kg). General obesity was defined as a BMI of ≥28.0 kg/m^2^ (36, 37). Abdominal obesity was defined as a WC of ≥90 cm in men and ≥85 cm in women (38). BFP was calculated using the following formula: Male: BFP = (1.20 × BMI + 0.23 × age − 5.4) × 100%; Female: BFP = (1.20 × BMI + 0.23 × age − 6.48) × 100% (39). Based on a relevant study (29), BFP obesity was defined as a BFP of ≥30% in men and ≥42% in women.

### 2.5 Assessment of covariates

To minimize the effect of potential confounders, statistical models were adjusted for factors, such as demographic characteristics, lifestyle habits, and health status. The demographic characteristics included in the analysis were as follows: age (18–60 years or ≥60 years), sex (male or female), marital status (married or other), and socioeconomic status (SES). The following lifestyle factors were included: smoking status (current smokers, former smokers, or never-smokers), drinking status (current drinkers, former drinkers, or never-drinkers), exercise habits (exercise currently, exercised previously, or exercised never), and weight control (yes or no). Regarding health status, the following common chronic diseases were included: hypertension (yes or no), diabetes (yes or no), heart disease (yes or no), cardiovascular disease (yes or no), and dyslipidaemia (yes or no). SES was evaluated by asking participants whether they (1) received secondary school education or higher, (2) were born in an urban area, and (3) earned at least 3,000 Chinese Yuan per month (40).

### 2.6 Statistical analyses

EpiData (version 3.1) was used for data entry and documentation, and Statistical Product and Service Solutions (version 27.0) was used for data processing and analysis. Descriptive statistics were calculated for general demographic characteristics, NL, and obesity. Categorical data are presented as frequency and percentage values. The chi-square test was performed to compare the correlations between NL levels and obesity types across the subgroups based on demographic characteristics. A binary logistic regression analysis was performed to investigate the correlation between NL and obesity in adults. Because the linear correlation between NL and obesity was unclear, tests for linearity in trends across quartiles were performed for the NL quartiles. The effect of overall NL on the risk of obesity was determined through subgroup analyses by age, sex, marital status, smoking status, and drinking status. Furthermore, the effects of interactions between these factors and NL levels on obesity were investigated. Between-group differences were considered to be statistically significant at *p* < 0.05.

## 3 Results

Table 1 presents the characteristics of the study population. This study included 2,156 individuals. Among them, 61.1% were aged >60 years and 61.1% were women. Moreover, 19.6% had the highest SES, and 78% were married. More than half were never-smokers (72.4%) or never-drinkers (63.6%), and approximately one-third excursed currently (28.5%), and controlled their weight (25.4%). Of the participants, 39.3% had hypertension, 12.4% had diabetes, 18.7% had heart disease, 16.4% had cardiovascular disease and 14.3% had dyslipidemia. The prevalence estimates of general obesity, abdominal obesity, and BFP obesity were 17.3, 57.9, and 52.2%, respectively.

**Table 1:** Characteristics of the study population.

Variable	*N* (%)	General obesity *n* (%)	*χ* ^2^	Abdominal obesity *n* (%)	*χ* ^2^	Body fat percentage obesity *n* (%)	*χ* ^2^
Total	2,156	373(17.3)		1,248(57.9)		1,126(52.2)	
Age			0.906		96.240***		307.141***
18–59	839(38.9)	137(16.3)		376(44.8)		240(28.6)	
60+	1,317(61.1)	236(17.9)		872(66.2)		886(67.3)	
Sex			6.101*		0.022		924.459***
Male	839(38.9)	124(14.8)		484(57.7)		782(93.2)	
Female	1,317(61.1)	249(18.9)		764(58.0)		344(26.1)	
Marital status			5.569*		8.655**		26.063***
Married	1,681(78.0)	308(18.3)		1,001(59.5)		927(55.1)	
Other	475(22.0)	65(13.7)		247(52.0)		199(41.9)	
SES			37.76***		46.107***		76.947***
0	741(34.4)	161(21.7)		473(63.8)		417(56.3)	
1	454(21.1)	98(21.6)		293(64.5)		268(59.0)	
2	539(25.0)	72(13.4)		256(47.5)		194(36.0)	
3	422(19.6)	42(10.0)		226(53.6)		247(58.5)	
Smoking status			1.305		3.117		404.050***
Current smoker	397(18.4)	61(15.4)		224(56.4)		342(86.1)	
Former smoker	198(9.2)	36(18.2)		126(63.6)		177(89.4)	
Never smoker	1,561(72.4)	61(15.4)		898(57.5)		607(38.9)	
Drinking status			8.107*		11.620**		203.625***
Current drinker	538(25.0)	74(13.8)		296(55.0)		385(71.6)	
Former drinker	247(11.5)	53(21.5)		167(67.6)		184(74.5)	
Never drinker	1,371(63.6)	246(17.9)		785(57.3)		557(40.6)	
Exercise habits			9.409**		6.852*		18.080
Exercise currently	1,284(28.5)	200(15.6)		748(58.3)		713(55.5)	
Exercised previously	244(34.0)	40(16.4)		123(50.4)		102(41.8)	
Exercised never	628(37.5)	133(21.2)		377(60.0)		311(49.5)	
Weight control			16.343***		1.213		6.549*
Yes	546(25.4)	125(22.9)		327(59.9)		259(47.4)	
No	1,605(74.6)	246(15.3)		918(57.2)		863(53.8)	
Hypertension			62.075***		149.992***		148.452***
Yes	846(39.3)	214(25.3)		627(74.1)		546(41.7)	
No	1,309(60.7)	159(12.1)		621(47.4)		580(68.6)	
Diabetes			21.430***		46.293***		16.992***
Yes	267(12.4)	73(27.3)		206(77.2)		171(64.0)	
No	1888(87.6)	300(15.9)		1,024(55.2)		955(50.6)	
Heart disease			19.462***		52.121***		47.82***
Yes	403(18.7)	100(24.8)		298(73.9)		273(67.7)	
No	1751(81.3)	273(15.6)		950(54.3)		852(48.7)	
Stroke/cardiovascular disease			1.410		24.453***		41.031***
Yes	354(16.4)	69(19.5)		247(69.8)		240(67.8)	
No	1801(83.6)	304(16.9)		1,001(55.6)		886(49.2)	
Dyslipidemia			22.997***		48.699***		24.894***
Yes	309(14.3)	83(26.9)		235(54.9)		202(65.4)	
No	1846(85.7)	290(15.7)		1,013(76.1)		924(50.1)	

General obesity, a BMI of ≥28 kg/m^2^; abdominal obesity, a waist circumference of ≥90 cm in men and ≥85 cm in women; body fat percentage obesity, a body fat percentage of ≥30% in men and ≥42% in women.

Table 2 presents the correlation between NL and obesity prevalence. The prevalence of general obesity (20.0%), abdominal obesity (64.1%), and BFP obesity (62.4%) were the highest among individuals with NL levels in the lowest quartile. As the level of NL increased, the prevalence of obesity decreased (*p* < 0.05). This correlation was applicable to both the cognition and skills domains of the NL.

**Table 2:** Correlation between nutrition literacy and obesity prevalence.

Variable	General obesity	*χ* ^2^	*p*-value	Abdominal obesity	*χ* ^2^	*p*-value	Body fat percentage obesity	*χ* ^2^	*p*-value
Nutrition literacy		21.010	<0.001		49.355	<0.001		73.995	<0.001
Q1	120(20.0)			384(64.1)			374(62.4)		
Q2	120(21.4)			356(63.3)			323(57.5)		
Q3	74(14.3)			293(56.7)			244(47.2)		
Q4	59(12.3)			215(45.0)			185(38.7)		
Nutrition cognition		11.970	0.007		41.098	<0.001		64.800	<0.001
Q1	147(20.0)			475(64.5)			438(59.5)		
Q2	91(19.2)			290(61.3)			278(58.8)		
Q3	74(15.4)			265(55.2)			233(48.5)		
Q4	61(13.1)			218(46.7)			177(37.9)		
Nutrition skills		17.962	<0.001		41.504	<0.001		59.650	<0.001
Q1	131(19.0)			403(63.5)			393(61.9)		
Q2	113(19.9)			361(63.4)			319(56.1)		
Q3	73(14.8)			271(54.9)			229(46.4)		
Q4	56(12.2)			213(46.5)			185(40.4)		

General obesity, a BMI of ≥28 kg/m^2^; abdominal obesity, a waist circumference of ≥90 cm in men and ≥85 cm in women; body fat percentage obesity, a body fat percentage of ≥30% in men and ≥42% in women.

Table 3 presents the results of the logistic regression performed to identify the correlation between NL and obesity. A model adjusted for all covariates revealed that participants with total NL levels in the highest quartile had low odds of general obesity (OR: 0.66; 95% confidence interval [CI]: 0.43–0.99; *p* for trend =0.026), abdominal obesity (OR: 0.63; 95% CI: 0.46–0.87; *p* for trend = 0.005), and BFP obesity (OR: 0.55; 95% CI: 0.35–0.87; *p* for trend = 0.004). This correlation was applicable to the cognition domain of the NL-SF12. Participants with nutrition skills in the highest quartile also had low odds of abdominal obesity (OR: 0.70; 95% CI: 0.51–0.95; *p* for trend = 0.017) and BFP obesity (OR: 0.51; 95% CI: 0.33–0.80; *p* for trend = 0.002); however, the association with general obesity was non-significant (OR: 0.68; 95% CI: 0.45–1.03; *p* for trend = 0.056).

**Table 3:** Results of a logistic regression analysis performed to identify the correlation between nutrition literacy and obesity.

Variable	General obesity OR (95%CI)	*p* for trend	Abdominal obesity OR (95%CI)	*p* for trend	Body fat percentage obesity OR (95%CI)	*p* for trend
Nutrition literacy		0.026		0.005		0.004
Q1	Reference		Reference		Reference	
Q2	1.09(0.80,1.49)		1.10(0.85,1.43)		0.89(0.64,1.26)	
Q3	0.69(0.48,1.00)		0.94(0.70,1.25)		0.55(0.36,0.82)**	
Q4	0.66(0.43,0.99)*		0.63(0.46,0.87)**		0.55(0.35,0.87)*	
Nutrition cognition		0.032		0.007		0.015
Q1	Reference		Reference		Reference	
Q2	0.97(0.70,1.32)		0.96(0.74,1.25)		1.20(0.84,1.70)	
Q3	0.78(0.54,1.11)		0.88(0.67,1.17)		0.68(0.46,1.01)	
Q4	0.66(0.45,0.97)*		0.65(0.48,0.86)**		0.60(0.40,0.93)*	
Nutrition skills		0.056		0.017		0.002
Q1	Reference		Reference		Reference	
Q2	1.04(0.76,1.41)		1.19(0.92,1.53)		0.85(0.61,1.19)	
Q3	0.77(0.54,1.11)		0.90(0.68,1.20)		0.61(0.41,0.91)*	
Q3	0.68(0.45,1.03)		0.70(0.51,0.95)*		0.51(0.33,0.80)**	

General obesity, a BMI of ≥28 kg/m^2^; abdominal obesity, a waist circumference of ≥90 cm in men and ≥85 cm in women; body fat percentage obesity, a body fat percentage of ≥30% in men and ≥42% in women.

All models were adjusted for age, sex, marital status, socioeconomic status (SES), smoking status, drinking status, exercise habits, weight control, hypertension, diabetes, heart disease, cardiovascular disease and dyslipidemia. SES was calculated as a continuous variable by means of education level, monthly income, and birthplace. The total SES score ranged from 0 to 3, with a higher score indicating a higher SES level. *p < 0.05, **p < 0.01, and ***p < 0.001.

Table 4 presents the associations between NL and obesity in the subgroups. High levels of NL were significantly associated with low odds of abdominal obesity and BFP obesity in participants aged 18–59 years (OR: 0.54 [95% CI: 0.31–0.96] and 0.35 [95% CI: 0.15–0.83], respectively), but not in those aged ≥60 years (OR: 0.71 [95% CI: 0.47–1.09] and 0.58 [95% CI: 0.33–1.04], respectively). Age exerted no significant effect on the association between a high level of NL and a low odd of general obesity (*p* > 0.05). Similar results were obtained for marital status. High levels of NL were significantly and negatively correlated with low odds of general obesity in women (OR: 0.42; 95% CI: 0.24–0.72), never-smokers (OR: 0.53; 95% CI: 0.32–0.87), and never-drinkers (OR: 0.45; 95% CI: 0.26–0.79), but not in men, smokers, or drinkers (*p* > 0.05). Similar results were obtained for abdominal obesity and BFP obesity. The interactions between NL and age exerted significant effects on abdominal obesity and BFP obesity (*p* for interaction = 0.019 and 0.007, respectively), but not on general obesity (*p* for interaction = 0.329). The interactions between NL and sex, and smoking status exerted significant effects on general obesity and abdominal obesity (*p* for interaction <0.05 for both), but not on BFP obesity (*p* for interaction >0.05). Furthermore, the interactions between NL and drinking status exerted significant effects on abdominal obesity (*p* for interaction =0.035), but not on general obesity and BFP obesity (*p* for interaction >0.05 for both). However, the interactions of NL with marital status exerted no significant effect on general obesity, abdominal obesity, or BFP obesity (*p* for interaction >0.05 for all).

**Table 4:** Results of subgroup analyses and interaction tests for the correlation of nutrition literacy with obesity.

Subgroups	Variables	General obesity OR (95%CI)	*p* for interaction	Abdominal obesity OR (95%CI)	*p* for interaction	Body fat percentage obesity OR (95%CI)	*p* for interaction
Age			0.329		0.019		0.007
18–59	Nutrition literacy (Q1)						
	Q2	0.87(0.48,1.59)		1.04(0.61,1.76)		0.42(0.20,0.88)*	
	Q3	0.58(0.31,1.11)		0.79(0.46,1.34)		0.25(0.11,0.56)***	
	Q4	0.54(0.27,1.09)		0.54(0.31,0.96)*		0.35(0.15,0.83)*	
60+	Nutrition literacy (Q1)						
	Q2	1.13(0.78,1.62)		1.07(0.79,1.45)		1.04(0.71,1.53)	
	Q3	0.68(0.42,1.09)		1.01(0.70,1.47)		0.65(0.39,1.08)	
	Q4	0.71(0.41,1.22)		0.71(0.47,1.09)		0.58(0.33,1.04)	
Sex			0.001		<0.001		0.642
Male	Nutrition literacy (Q1)						
	Q2	1.30(0.74,2.30)		0.92(0.62,1.37)		1.31(0.39,4.45)	
	Q3	0.99(0.52,1.88)		0.75(0.48,1.16)		1.17(0.36,3.82)	
	Q4	1.47(0.73,2.93)		0.84(0.51,1.39)		1.71(0.46,6.42)	
Female	Nutrition literacy (Q1)						
	Q2	1.02(0.70,1.48)		1.31(0.91,1.88)		0.89(0.63,1.28)	
	Q3	0.57(0.35,0.91)*		1.22(0.81,1.82)		0.49(0.31,0.77)**	
	Q4	0.42(0.24,0.72)**		0.61(0.40,0.94)*		0.45(0.27,0.75)**	
Marital status			0.575		0.861		0.758
Married	Nutrition literacy (Q1)						
	Q2	1.06(0.75,1.49)		1.03(0.77,1.39)		0.81(0.54,1.21)	
	Q3	0.67(0.44,1.01)		0.90(0.65,1.25)		0.50(0.30,0.81)**	
	Q4	0.62(0.39,1.00)		0.59(0.42,0.85)**		0.46(0.26,0.81)**	
Other	Nutrition literacy (Q1)						
	Q2	1.22(0.55,2.69)		1.54(0.84,2.81)		1.17(0.60,2.30)	
	Q3	0.80(0.31,2.05)		1.22(0.61,2.41)		0.70(0.30,1.63)	
	Q4	0.94(0.35,2.53)		0.84(0.40,1.73)		0.95(0.39,2.32)	
Smoking status			0.024		0.005		0.477
Current smoker	Nutrition literacy (Q1)						
	Q2	2.01(0.87,4.62)		1.08(0.61,1.90)		1.57(0.46,5.30)	
	Q3	2.12(0.82,5.47)		1.22(0.63,2.35)		1.44(0.39,5.41)	
	Q4	1.55(0.53,4.59)		0.72(0.35,1.49)		0.97(0.23,4.07)	
Former smoker	Nutrition literacy (Q1)						
	Q2	0.79(0.22,2.83)		0.85(0.32,2.21)		0.71(0.03,16.51)	
	Q3	0.46(0.12,1.84)		0.52(0.18,1.47)		0.24(0.01,6.44)	
	Q4	1.21(0.31,4.77)		1.30(0.39,4.32)		2.35(0.06,87.40)	
Never smoker	Nutrition literacy (Q1)						
	Q2	1.04(0.73,1.48)		1.19(0.86,1.64)		0.88(0.61,1.27)	
	Q3	0.58(0.38,0.91)*		0.99(0.70,1.42)		0.48(0.30,0.75)**	
	Q4	0.53(0.32,0.87)*		0.60(0.41,0.88)*		0.50(0.30,0.83)**	
Drinking status			0.060		0.035		0.713
Current drinker	Nutrition literacy (Q1)						
	Q2	0.92(0.44,1.94)		0.90(0.54,1.52)		0.96(0.38,2.42)	
	Q3	0.81(0.37,1.78)		0.99(0.58,1.69)		0.74(0.29,1.89)	
	Q4	1.35(0.55,3.31)		0.77(0.41,1.44)		0.95(0.31,2.88)	
Former drinker	Nutrition literacy (Q1)						
	Q2	1.13(0.43,2.94)		0.72(0.29,1.75)		0.31(0.06,1.71)	
	Q3	0.57(0.17,1.90)		0.43(0.15,1.19)		0.10(0.02,0.71)*	
	Q4	1.51(0.48,4.79)		0.69(0.24,2.01)		0.40(0.04,3.88)	
Never drinker	Nutrition literacy (Q1)						
	Q2	1.16(0.79,1.68)		1.32(0.95,1.84)		0.89(0.61,1.31)	
	Q3	0.73(0.45,1.17)		1.05(0.72,1.54)		0.55(0.34,0.90)*	
	Q4	0.45(0.26,0.79)**		0.60(0.40,0.90)*		0.46(0.27,0.79)**	

General obesity, a BMI of ≥28 kg/m^2^; abdominal obesity, a waist circumference of ≥90 cm in men and ≥85 cm in women; body fat percentage obesity, a body fat percentage of ≥30% in men and ≥42% in women. All models were adjusted for age, sex, marital status, socioeconomic status (SES), smoking status, drinking status, exercise habits, weight control, hypertension, diabetes, heart disease, cardiovascular disease and dyslipidemia. SES was calculated as a continuous variable by means of education level, monthly income, and birthplace. The total SES score ranged from 0 to 3, with a higher score indicating a higher SES level. *p < 0.05, **p < 0.01, and ***p < 0.001.

## 4 Discussion

This study explored the correlation of NL with general obesity, abdominal obesity, and BFP obesity in adults. Our findings revealed that adults with high levels of NL had low odds of general obesity, abdominal obesity, and BFP obesity. Moreover, the interactions between NL and age, sex, smoking status, and drinking status exerted significant effects on obesity. For the prevention and management of obesity in adults, policymakers and community health workers should incorporate NL assessment and improvement into relevant strategies. Furthermore, to reduce the prevalence of obesity among adults, their NL levels should be enhanced and targeted interventions that integrate sex, age, and lifestyle factors should be developed.

In this study, we found a negative correlation between NL and obesity in adults. This finding is supported by those of other studies. For example, Gong (41) demonstrated that individuals with poorer nutrition knowledge and behavior had a higher risk of obesity. People with higher levels of NL are more likely to choose low-calorie, high-nutrient-density foods (42); thus, they have a lower risk of obesity. Yang et al. (43) reported that individuals with higher levels of nutrition-related cognition are better equipped to interpret food labels and comprehend healthy eating guidelines, which help them make healthier dietary choices. A healthy diet can help control body weight and reduce abdominal fat and BFP (44, 45). Adults with higher NL levels are more likely to have a healthier lifestyle, characterized by factors such as superior sleep quality and regular exercise (46, 47), which, in turn, reduces the risk of obesity (48, 49). Higher NL levels are also associated with greater self-efficacy and more positive health attitudes (50), which help individuals to more effectively cope with stress and mood swings, thereby reducing the risk of overeating (51). Enhancing NL levels may mitigate the risks of various types of obesity in Chinese adults.

This study further explored the association of individuals’ NL scores in both the cognition and skill domains of the NL with their odds of general obesity, abdominal obesity, and BFP obesity. The results indicated that NL scores in the cognition domain were negatively associated with the odds of general obesity, abdominal obesity, and BFP obesity in adults. By contrast, NL scores in the skill domain of the NL were negatively associated with the odds of abdominal obesity and BFP obesity, but not general obesity. This discrepancy may be attributable to the fact that abdominal obesity and BFP obesity indicate the distribution of body fat, whereas BMI is an overall indicator of body weight (52). Thus, BMI may be an inadequate reflection of health. Qi et al. (42) described that individuals with high levels of nutrition-related skills effectively manage their diets—for example, by avoiding fast food, which influences the quality and quantity of diet, thereby reducing the risks of abdominal obesity and BFP obesity. However, these skills exert limited effects on BMI (53, 54). Therefore, although high levels of NL may not lead to significant changes in body weight, NL leads to marked improvements in diet quality, which reduces abdominal fat and BFP. In summary, nutrition-related skills may exert direct effects on the odds of abdominal obesity and BFP obesity, whereas BMI, which is a crude indicator, fails to effectively capture these nuances. Our findings are also supported by those of a cross-sectional study, which demonstrated associations of overweight and obesity with functional NL, but not with interactive and critical skill-related NL (17). Public health workers should improve the NL levels of adults through education, encouraging them to acquire nutrition-related knowledge and skills and incorporate these into their daily lives. For example, by perusing nutritional information on food labels and evaluating dietary habits, the risk of obesity can be reduced in adults.

In the present study, subgroup analyses and interaction tests revealed potential confounders influencing the correlation between NL and obesity: age, sex, smoking status, and drinking status. Gao et al. (55) suggested that the level of NL is typically higher in women than in men. Therefore, women maintain healthier diets (56), which reduces the risk of obesity (57). Research has consistently demonstrated that the prevalence of general obesity is lower among women than among men (58). Between-sex differences have been noted also in body fat distribution; men are more likely than women to accumulate fat in the abdomen (59). Consequently, sex may influence the correlation of NL with both general obesity and abdominal obesity. Furthermore, women typically exhibit a higher BFP than men (60). Thus, sex influences the correlation between NL and BFP obesity. Smoking increases BMI and visceral fat content, thereby increasing the risks of general obesity and abdominal obesity (61, 62). However, smoking is unlikely to be a significant risk factor for BFP obesity (63). Alcohol consumption may be associated with increased calorie intake and thus weight gain and abdominal fat accumulation (64, 65). However, a systematic review by Bendsen et al. (66) showed that alcohol consumption was positively associated with abdominal obesity rather than general obesity. In addition, alcohol consumption is not directly associated with BFP obesity (67). Individuals with higher NL levels are more healthy aware and better able to control alcohol consumption (30), which reduces the risks of abdominal obesity. Younger people tend to have higher levels of education and NL than older people (16); thus, the association between NL and obesity is more pronounced in the younger population than in the older population. Fat distribution is influenced by age. Older adults exhibit greater muscle loss (64). Thus, older adults are at higher risks of abdominal obesity and BFP obesity (68, 69). However, BMI may not accurately reflect fat distribution changes. In summary, the negative correlation between NL and obesity is influenced by sex, smoking habit, alcohol consumption, and age. These findings indicate that improving NL can reduce the odds of various types of obesity. Thus, public health practitioners should develop targeted interventions incorporating demographic and lifestyle factors to reduce the risk of obesity.

This study has some strengths. This study comprehensively evaluated the relationship between NL and multiple types of obesity in adults. Furthermore, weight, height, and WC were all measured by trained researchers; no self-reported data were used for these parameters, thereby reducing the misclassification bias associated with self-reported measurements. However, this study also has some limitations. First, although the cross-sectional design of this study facilitated the investigation of the correlation between NL and obesity, it precluded the establishment of any causal relationships. In the future, longitudinal study designs can be conducted to further explore the causal association between NL and obesity in adults. Second, self-reported information on covariates might have introduced bias during data collection. Third, the survey did not inquire about conditions such as pregnancy, breastfeeding, menstrual cycle, menopause status, disease-related body edema, or medication usage that might influence weight and fat distribution, which could potentially impact the study results. Finally, our study sample was recruited from only one city in Anhui Province. Therefore, caution should be exercised when extrapolating the results to other ethnic groups.

## 5 Conclusion

This study revealed that higher levels of NL were strongly correlated with lower odds of general obesity, abdominal obesity, and BFP obesity. These correlations varied depending on sex, age, smoking status, and drinking status and underscore the importance of NL in obesity prevention. Thus, public health policies should be formulated to improve NL and thus mitigate obesity. Furthermore, community health workers should tailor nutrition education and skill training programs to specific demographic profiles, considering factors such as sex, age, and obesity type. This targeted approach can precisely and effectively reduce the risk of obesity.

## Data Availability

The raw data supporting the conclusions of this article will be made available by the authors, without undue reservation.
